# A Recurrent Motif: Diversity and Evolution of ShKT Domain Containing Proteins in the Vampire Snail *Cumia reticulata*

**DOI:** 10.3390/toxins11020106

**Published:** 2019-02-12

**Authors:** Marco Gerdol, Manuela Cervelli, Paolo Mariottini, Marco Oliverio, Sébastien Dutertre, Maria Vittoria Modica

**Affiliations:** 1 Department of Life Science, Trieste University, I-34127 Trieste, Italy; mgerdol@units.it; 2 Department of Science, Roma Tre University, I-00143 Rome, Italy; manuela.cervelli@uniroma3.it (M.C.); paolo.mariottini@uniroma3.it (P.M.); 3 Department of Biology and Biotechnologies “Charles Darwin”, La Sapienza University of Rome, I-00185 Rome, Italy; marco.oliverio@uniroma1.it; 4 UMR5247, University of Montpellier, CC 1703-Place Eugène Bataillon, 34095 Montpellier, France; 5 Department of Integrative Marine Ecology, Stazione Zoologica Anton Dohrn, I-80121 Naples, Italy

**Keywords:** ShKT, CRISP, Astacin, Gastropoda, haematophagy, parasitism, domain duplication, gene duplication

## Abstract

Proteins of the ShK superfamily are characterized by a small conserved domain (ShKT), first discovered in small venom peptides produced by sea anemones, and acting as specific inhibitors of voltage-dependent and calcium-activated K^+^ channels. The ShK superfamily includes both small toxic peptides and larger multifunctional proteins with various functions. ShK toxins are often important components of animal venoms, where they perform different biological functions including neurotoxic and immunosuppressive effects. Given their high specificity and effectiveness, they are currently regarded as promising pharmacological lead compounds for the treatment of autoimmune diseases. Here, we report on the molecular analysis of ShKT domain containing proteins produced by the Mediterranean vampire snail *Cumia reticulata*, an ectoparasitic gastropod that feeds on benthic fishes. The high specificity of expression of most ShK transcripts in salivary glands identifies them as relevant components of *C. reticulata* venom. These ShK proteins display various structural architectures, being produced either as single-domain secretory peptides, or as larger proteins combining the ShKT with M12 or CAP domains. Both ShKT-containing genes and their internal ShKT domains undergo frequent duplication events in *C. reticulata*, ensuring a high level of variability that is likely to play a role in increasing the range of their potential molecular targets.

## 1. Introduction

The ShK toxin (ShKT) is a potent potassium (K^+^) channel blocker, which was originally isolated from the Caribbean sea anemone *Stichodactyla helianthus* [1] and subsequently chemically synthesized [2]. The original ShKT is a peptide motif of 35 amino acid (aa) residues that includes six cysteines, which form three disulfide bonds with connectivity C1-C6, C2-C4 and C3-C5. Potassium channels are ubiquitous tetrameric membrane proteins that regulate membrane potential and calcium signaling in diverse cell types, including those involved in both innate and adaptive immune response. Given their widespread distribution and their central physiological role in all living organisms, K+ channels are the target of hundreds of toxins that generally contain between 18 and 60 residues, structured with 2-3 disulfide bonds, which confer structural stability and resistance to denaturation [3,4]. 

Since the discovery of ShK, a high number of such toxins from sea anemones have been characterized and have been assigned different names (e.g., stichotoxins, actitoxins or thalatoxins), depending on the species of origin [5,6,7]. A recent classification includes ShK in the sea anemone type 1 potassium channel toxin family [8]. This family includes 27–35 aa-long domains, mostly from sea anemones (Cnidaria, Anthozoa, Actiniaria), which interfere with binding of radiolabelled snake dendrotoxin to synaptosomal membranes and block currents through channels with various K_V_1 subunits and also intermediate conductance K(Ca) channels. Molecular modelling coupled with mutational analyses has identified Lys22 as a key residue for the blockage of the K^+^ channel, since this residue is able to penetrate and occlude the pore of the channel [9,10], while Ser20, Lys25 and Tyr23 have been demonstrated to be responsible for the binding of ShK to K_V_ channels in rat brain [3]. Since corresponding residues are conserved in other toxins (e.g., in scorpion toxins) and also involved in the same binding process, the dyad Lys22-Tyr23 is regarded to be essential for the binding of toxins to K_V_ channels [8]. 

The ShKT has a very high affinity (Ki ~10 pM) for K_V_1.3 channels but also displays high affinity for K_V_1.1, K_V_1.4 and K_V_1.6 channels present in brain and cardiac tissues [2,10]. Anyway, the therapeutic potential of ShKT is mostly linked to its ability to block K_V_1.3 channels. These can be considered as the functionally dominant channels in terminally differentiated effector memory (TEM) T cells, where they are crucial for activation. Since TEM cells are involved in multiple autoimmune conditions, K_V_1.3 has been considered a promising target for the treatment of T cell-mediated autoimmune diseases, and for the prevention of transplant rejection [11]. A number of ShK peptide analogues with increased affinity for K_V_1.3 channels have been synthetized [12,13,14,15,16,17], and in some cases these molecules have demonstrated their efficacy in animal models of human autoimmune diseases. Among them, the development of Dalazatide, the analogue ShK-186, has completed phase 1 preclinical trials, and shows a prolonged therapeutic efficacy that makes it a very promising treatment for a number of autoimmune diseases [9,18,19,20].

Other cnidarians possess proteins structurally similar to ShK; for example, the scyphozoan *Aurelia aurita* produces aurelin, which is not associated with nematocysts, but acts in innate immunity as an antimicrobial peptide [21]. The typical structural fold of ShKT defines an evolutionarily highly conserved protein motif that has also been found in a great number of multidomain proteins, both from animals and from plants [20]. These ShKT-domain containing proteins mostly comprise metalloproteases, but also prolyl-4-hydroxylases, tyrosinases, peroxidases, and oxydoreductases, depending on the structural organization of the polypeptide, which can combine multiple accessory domains (e.g., epidermal growth factor-like domains, thrombospondin-type repeats, or trypsin-like serine protease) together with the ShKT motif [22]. Several cases of proteins containing multiple consecutive ShKT domains have been reported, including the three-domain ShKT proteins of the cnidarian *Nematostella vectensis* [23] and the ShKT-domain containing proteins of roundworms (Nematoda), which currently constitute the largest known protein family with ShKT domains [20].

In the phylum Mollusca, the ShKT domain has also been reported in multidomain proteins, mostly metalloendopeptidases (170 Uniprot entries), none of which has been so far further investigated or functionally characterized. To date, secreted single-domain ShK toxins have been only reported from the Mediterranean vampire snail *Cumia reticulata* (Gastropoda, Colubrariidae), and identified as salivary gland (SG)-specific products based on transcriptomic evidence [24]. However, the same study also highlighted the presence of multidomain proteins where ShKT domains are associated either with CAP (cysteine-rich secretory proteins, antigen 5, and pathogenesis-related 1 proteins) or with M12 (astacin metalloprotease) domains. 

The association between CAP and ShkT domains is relatively common in nature, and it is found in more than 600 proteins currently deposited in public sequence databases, both from vertebrates and invertebrate organisms. Most notably, the ShKT motif is often embedded in the larger cysteine array of the CRISP domain, which takes its name from vertebrate cysteine-rich secretory proteins (CRISPs). These multifunctional proteins, which have been linked with the proper functioning of the mammalian reproductive system [25] are most commonly known for their role as cyclic nucleotide-gated ion channel inhibitors in the venom of snakes [26,27], lizards [28] and lampreys [29]. The MEROPS peptidase family M12 domain (InterPro: IPR001506) is the characterizing signature of astacins, a very large family of metalloendoproteases with widespread taxonomic distribution and diverse biological functions [30]. Astacin-like proteases often combine the M12 domain with several other N-terminal or C-terminal modules, with CUB being the most prevalent. M12-CUB proteases are found in a wide range of organisms, from vertebrates to the nematode *Caenorhabditis elegans*, where they form a largely expanded gene family [31]. About 900 out of the more than 10,000 known animal M12-domain containing proteins have ShKT modules in their C-terminal region. While this architecture is not found in vertebrates, M12-ShK proteins are found in various protostome and deuterostome invertebrate animals. 

Colubrariidae are haematophagous snails that feed on the blood of sleeping benthic fishes, mostly at night time [32,33,34]. After having reached a fish with their long proboscis, colubrariids pierce its skin (probably with the aid of a cytolytic secretion mainly consisting of coluporins [35]), and feed passively on its blood. Observations in nature and in aquariums suggest that the fish is reversibly anesthetized during feeding ([34] and unpublished observations); additionally the production of multiple anti-haemostatic compounds, which are fundamental for a haematophagous trophic strategy has been demonstrated [24,36]. 

While some ShKT domain-containing proteins have been reported in a previous transcriptomic analysis on the secretory tissues of the foregut in *C. reticulata* (including both the salivary glands, and the glandular mid-oesophageal region, which is a peculiar trait of Colubrariidae) [24], we provide here an improved characterization of their full-length sequences, with an in silico assessment of their correctness. We also describe their evolutionary history and patterns of expression in detail, and discuss their possible involvement in the haematophagus feeding habit of this vampire snail.

## 2. Results

### 2.1. ShK-Like Proteins

**Figure 1:**
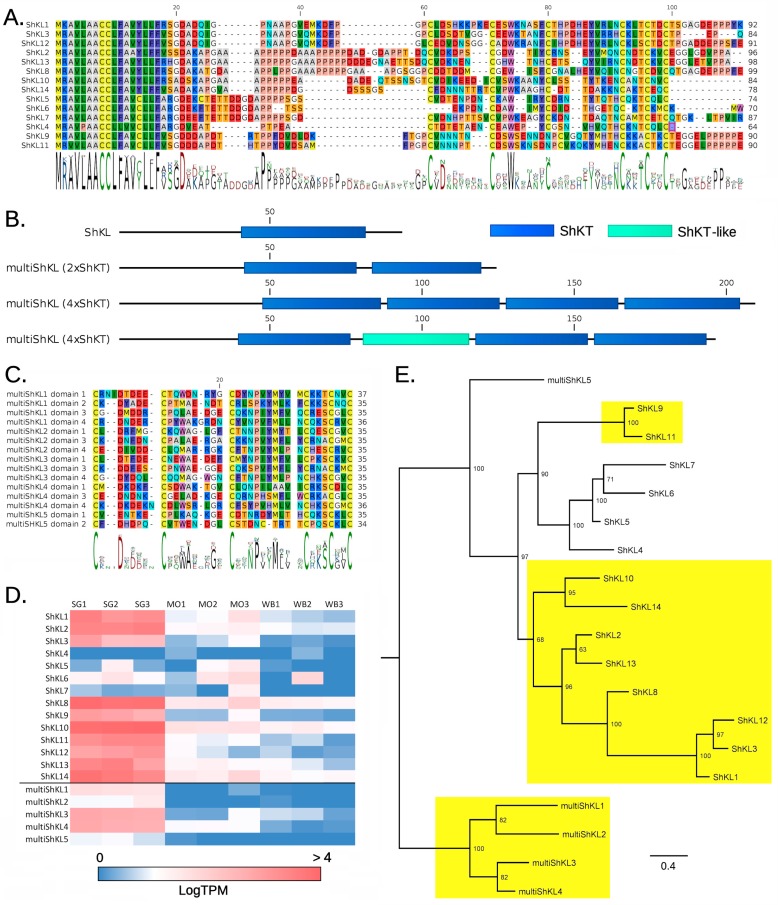
(**A**) Multiple sequence alignment of the 14 ShKL proteins from *Cumia reticulata*. The sequence consensus is indicated as a sequence logo below the alignment. (**B**) Schematic representation of ShKL and multiShKL proteins. Each domain is shown with a box of different colour. ShKT and ShKT-like: ShK toxin and ShK toxin-like domains; note that multiShKL proteins can either contain four canonical ShKT domains (as in the case of multiShKL 1) or a three canonical plus a ShKT-like domain (as in the case of multiShKL 2, 3 and 4). (**C**) Multiple sequence alignment of ShKT domains from ShKL proteins. The domain consensus is indicated as a sequence logo below the alignment. (**D**) Expression pattern of ShKL transcripts, based on Modica et al. [24]. Expression levels are given as Log(TPM). SG: salivary gland; MO: mid-oesophageal gland; WB: whole body. (**E**) Bayesian analysis of *C. reticulata* ShKL and multiShKL proteins. SG-specific genes are highlighted with a yellow background. Posterior probability support values are shown for each node.

We identified 14 ShK-like (ShKL) proteins in the transcriptome of *C. reticulata* (henceforth referred to as CreShKL), sharing a nearly identical 22 aa-long signal peptide, followed by a low complexity region of variable length (8–30 residues), the ShKT domain and an optional short low complexity C-terminal extension (Figure 1, panel A). Both the N-terminal and C-terminal low complexity regions were enriched in proline residues, and in some cases (ShKL 2, 8, 10, 13 and 14) the N-terminal region contained the sequence stretch AAAPPPPP, repeated one or two times. *C. reticulata* ShKL protein precursors ranged in size from 64 (ShKL4) to 99 (ShKL8) aa residues. In all but one cases, these proteins displayed a well-recognizable ShKT motif, with 6 highly conserved cysteine residues (Figure 1, panel A). The only exception was the ShKL 6 peptide, which critically lacks Cys1. 

We also identified multi-domain secretory ShKT-domain-containing proteins (here named multiShKL); a single full-length sequence encoded a protein with 2 ShKT domains (multiShKL 5) whereas four mRNAs encoded longer proteins characterized by four ShKT modules (multiShKL 1-4) (Figure 1, panel B). The length of such proteins ranged from 123 (multiShKL 5) to 208 (multiShKL 1) aa residues. Like their shorter counterparts, these polypeptides had a low-complexity, proline-rich N-terminal region linking the signal peptide region to the first cysteine-rich array. Interestingly, while the majority of the ShKT domains were canonical (Figure 1, panel C), the second ShKT domain of multiShKL 2, 3 and 4 lacked Cys1 and Cys6, exactly as in the last ShKT module of some CreM12-ShK proteins (see below).

CreShKL were massively expressed in SG, reaching values > 10,000 TPMs for ShKL 8 and 10 (Figure 1, panel D). The cumulative expression of all CreShKL sequences reached 32,000–40,000 TPMs in the three SG biological replicates, indicating that 3.2–4% of the global transcriptional effort of the SG tissue was invested in the production of mRNAs encoding these short secretory proteins. Eleven out of the 15 ShKL proteins showed extreme SG specificity, being expressed hundreds of times more than in the mid-oesophageal gland (MO) or whole body (WB), while ShKL 4, 5, 6 and 7 were expressed at nearly negligible levels in all tissues, similar to multiShK5, which contains two ShKT domains (Figure 1, panel D). The four multiShKL with four ShKT modules also displayed strong tissue specificity and either moderate (multiShKL 1 and 2, < 20 TPM) or strong (multiShKL 3 and 4, > 300 TPM) expression in SG (Figure 1, panel D).

A Bayesian analysis clearly distinguished the 4-domain multiShKL from the other proteins, placing multiShKL 5 (which contains two domains) in a basal position within the clade including all single-domain ShKL proteins (Figure 1, panel E). Curiously, the 4 sequences lacking tissue specificity were included in a well-supported subclade (posterior probability = 100%). 

### 2.2. CAP-ShKT Proteins

**Figure 2:**
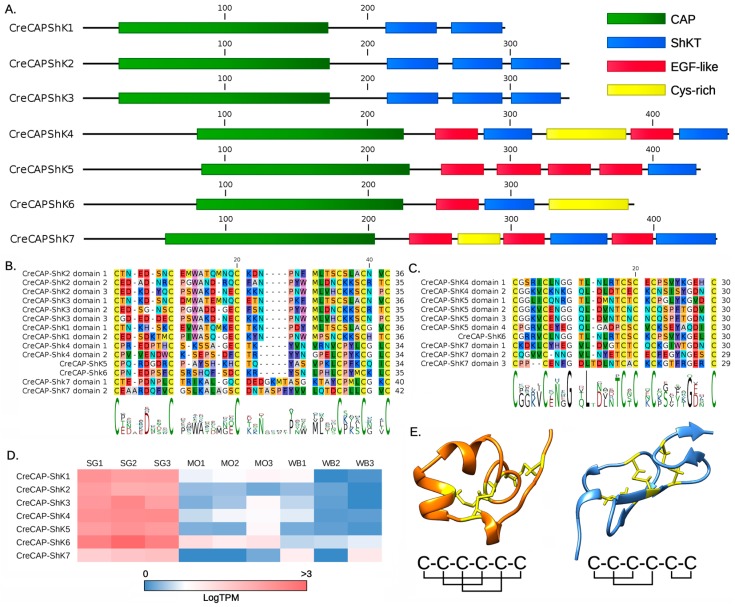
(**A**) Schematic representation of CreCAP-ShK proteins. Each domain is shown as a box of different color. CAP: cysteine-rich secretory proteins, antigen 5, and pathogenesis-related 1 proteins domain; ShKT: ShK toxin domain; EGF-like: epidermal growth factor-like domain; Cys-rich: other uncharacterized cysteine-rich motifs. (**B**) Multiple sequence alignment of ShKT domains from CreCAP-ShK proteins. The domain consensus is indicated as a sequence logo below the alignment. (**C**) Multiple sequence alignment of EGF-like domains from CreCAP-ShK proteins. The domain consensus is indicated as a sequence logo below the alignment. (**D**) Expression pattern of CreCAP-ShK transcripts, based on Modica et al. [24]. Expression levels are given as Log(TPM). SG: salivary gland; MO: mid-oesophageal gland; WB: whole body. (**E**) Structure and disulfide connectivity of ShKT (left) and EGF-like (right) domains, as exemplified by crystal structures of the *Stichodactyla helianthus* ShK toxin (PDB ID: 1ROO) and the human factor VII (PDB ID: 1BF9).

Here we report the identification of seven distinct CAP-ShK proteins specifically expressed in the SG tissue, which we will henceforth name CreCAP-ShK. Although all these molecules have an N-terminal signal peptide for secretion and display a well-recognizable CAP domain, they differ markedly in the length and organization of their C-terminal cysteine-rich region. Based on these architectural features, we defined two distinct sequence groups, i.e., CreCAP-ShK type I (CreCAP-ShK 1, 2 and 3) and II (CreCAP-ShK 4, 5, 6 and 7) proteins. While the former group only contains canonical ShKT domains, the latter contain poorly recognizable ShKT domains combined with EGF-like domains and other uncharacterized cysteine motifs (Figure 2, panel A). This classification is consistent with pairwise sequence similarity metrics, as type I sequences share >70% sequence identity, and type II sequences (with the exception of the more divergent CreCAP-ShK 7) share >45% sequence identity.

The length of CreCAP-ShK proteins is quite variable, ranging from 295 (CreCAP-ShK 1) to 452 (CreCAP-ShK 4) aa residues. These differences are ascribable both to the different organization of the C-terminal cysteine-rich region (Figure 2, panel A) and to the presence of a 30–50 aa long low-complexity spacer region, located N-terminal to the CAP domain in type II proteins. The detailed inspection of the cysteine-rich region of type I polypeptides revealed the presence of two (CreCAP-ShK 1) or three (CreCAP-ShK 2 and 3) consecutive repeats of the ShKT domain (Figure 2, panel B), in all cases recognizable with significant e-values (< 1E-3) with Hmmer. Among type II sequences, only CreCAP-ShK 7 displayed a recognizable ShKT domain whereas the other non-canonical domains, which differ from those identified in type I sequences in the length of the loop connecting Cys3 and Cys4 (Figure 2, panel B), were detected with the aid of a BLAST approach, as detailed in the Materials and Methods section.

The architecture of the C-terminal cysteine-rich region of CreCAP-ShK type II proteins is schematically represented in Figure 2, panel A. Besides ShKT-like motifs, they also presented EGF-like domains, composed by 30–40 aa and extremely common in animal proteins with diverse functions (nearly 90,000 EGF-like domain containing proteins are deposited in UniProtKB). This motif is characterized by a typical pattern of six (or eight) cysteines, with different sequence (Figure 2, panel C), disulfide connectivity and three-dimensional structure compared to ShKT (Figure 2, panel E). EGF-like and ShK-like domains are mixed with no apparent consensus in the four CreCAP-ShK type II proteins, being often interleaved with other unrecognizable arrays of 6 or 8 cysteine residues (Figure 2, panel A).

All CreCAP-ShK transcripts were expressed predominantly in SG, exceeding by >100-fold the transcriptional levels observed in MO and WB, where the expression was negligible (<10 TPM, but in most cases <1 TPM). However, the seven transcripts reached different expression values, with CreCAP-ShK 6 and 7 being the most (averaging ~850 TPM) and the least expressed (averaging ~25 TPM), respectively (Figure 2, panel D). 

**Figure 3:**
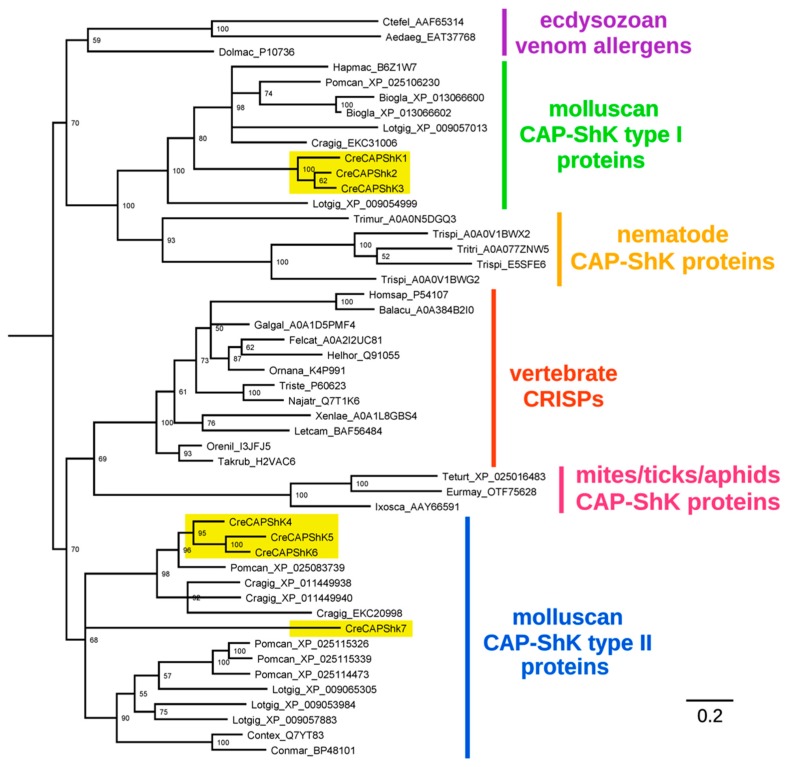
Bayesian analysis of CAP-ShK proteins and other related invertebrate proteins. CreCAP-ShK sequences are highlighted with a yellow background. Posterior probability support values are shown for each node, and nodes with low support (<50%) have been collapsed. Sequences are named as follows: the first three letters of the genus and species name, followed by the sequence accession ID.

We investigated whether the CAP-ShKT domain combination observed in *C. reticulata* could be considered as a lineage-specific innovation, evolved in the context of haematophagous feeding. The Bayesian analysis provided a clear response to this evolutionary question, placing CreCAP-ShK type I and II proteins in two distinct highly supported clades, both inferred to derive from ancestral genes which were likely already present in the latest common ancestor of all molluscs, given the presence of orthologous genes in modern non-predatory gastropods and bivalves alike (Figure 3).

The type I sequence clade also comprised several similar molluscan proteins with 1-4 canonical ShKT domains and with unknown function. This group notably included a partial protein isolated from the secretory glands of the octopus *Hapalochlaena maculosa* [37]. The type II sequence clade contained a heterogeneous group of sequences with variable, cysteine-rich C-terminal domains of different length, which may or may not include detectable ShKT domains, but often possessed recognizable EGF-like motifs. *Conus* Mr30 and Tex31 proteins were notably included within this clade, together with proteins from non-predatory gastropods and bivalves (Figure 3).

### 2.3. M12-ShKT Proteins

**Figure 4:**
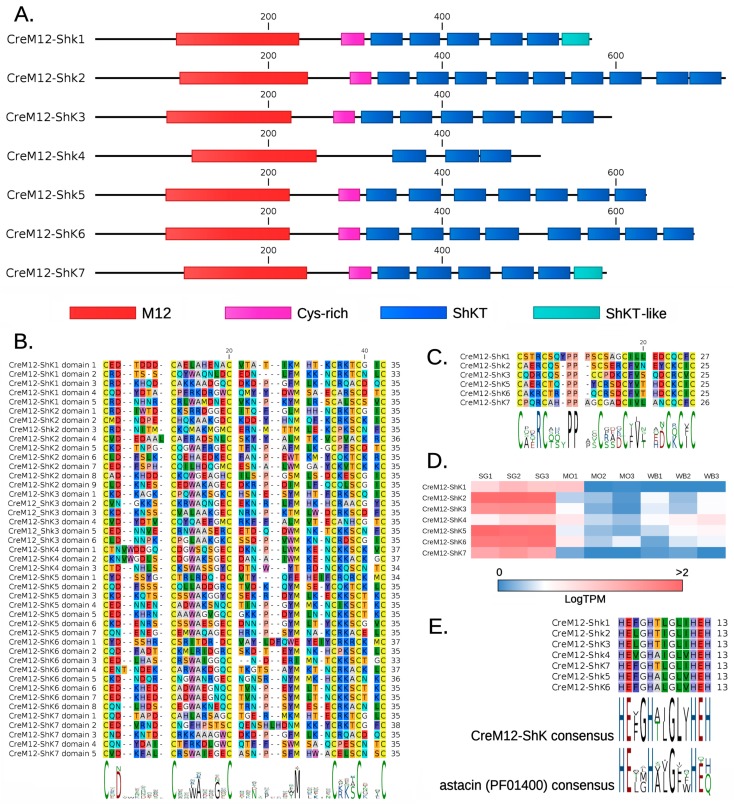
(**A**) Schematic representation of CreM12-ShK proteins. Each domain is shown as a box of different colour. M12: MEROPS peptidase family M12; ShKT and ShKT-like: ShK toxin and ShK toxin-like domains; Cys-rich: uncharacterized cysteine-rich motifs. (**B**) Multiple sequence alignment of ShKT domains from CreM12-ShK proteins. The domain consensus is indicated as a sequence logo below the alignment. (**C**) Multiple sequence alignment of the uncharacterized cysteine-rich motifs located in an N-terminal position compared to the first ShKT domain in CreM12-ShK proteins. The domain consensus is indicated as a sequence logo below the alignment. (**D**) Expression pattern of CreM12-ShK transcripts, based on Modica et al. [24]. Expression levels are given as Log(TPM). SG: salivary gland; MO: mid-oesophageal gland; WB: whole body. (**E**) Conservation of the zinc-binding motif in CreM12-ShK proteins and comparison with the consensus sequence from Pfam (PF01400).

We could identify seven transcripts encoding full-length M12-ShK proteins in *C. reticulata* (henceforth referred to as CreM12-ShK), but the size of this gene family is likely larger, as suggested by the detection of a number of partial contigs denoting fragmentation. The identified proteins shared an N-terminal signal peptide for secretion, followed by a 50–80 aa long uncharacterized region, and the M12 domain, which was always recognizable with respect to the Pfam astacin domain consensus (PF01400). This zinc-dependent functional domain displayed a good level of overall conservation in all seven sequences, in particular for the ion-binding active site exemplified by the consensus sequence HEXGHXXGLXHEH (Figure 4, panel E).

The precursor proteins sequences of CreM12-ShK varied from 512 (CreM12-ShK 4) to 725 (CreM12-ShK 2) aa residues. This size variation was due to the different number of ShKT motifs present, which range from 3 (in CreM12-ShK 4) to 9 in (CreM12-ShK 2) (Figure 4, panel A and B). Curiously, the first ShK module was preceded by an uncharacterized cysteine array containing seven conserved cysteine residues, with the single exception of CreM2-Shk 4 (Figure 4, panel C). Like multiShkL and CreCAP-ShK, also CreM12-ShK proteins contained a few unconventional ShKT domains: in detail, CreM12-ShK 1 and 7 lacked Cys1 and Cys6 (which are expected to be engaged in a bond). Although the ShKT domains were usually separated by short sequence linkers, in some cases (most notably in CreM12-ShK 6, between the ShKT domains 3 and 4) longer low-complexity sequence stretches were present.

M12-ShK mRNAs were mostly detected in SG, in five cases with values >100 fold higher than MO and WG. CreM12-ShK 1 and 4 were poorly expressed (<10 TPM) in all tissues, and only displayed moderate tissue specificity (about 3 times higher than the other tissues). The most highly expressed transcript, M12-ShK 2, averaged 160 TMP in SG. This value was just slightly higher than CreM12-ShK 5 and 6. CreM12-ShK 3 and 7 were expressed at moderate levels (30–50 TPM), but still displayed strong tissue specificity (Figure 4, panel D).

From an evolutionary perspective, gastropod M12-ShK proteins were highly diversified and pertained to different and distantly related lineages. Notably, the Bayesian analysis showed that molluscan sequences are quite divergent from those found in cnidarians and nematodes, whereas those from flatworms are intermixed with those from gastropods and bivalves (Figure 3). CreM12-ShK 4, the only protein containing only 3 ShKT domains and also one of the two lacking strong SG-specificity, was clearly related to orthologous metalloproteases found in all molluscs, regardless of the feeding habits of the species of origin. On the other hand, the other six M12-ShK sequences were grouped in a strongly supported monophyletic clade. 

## 3. Discussion

Although the ShKT domain is a rather common structural fold in the animal kingdom, it is often combined with other functional modules in large multidomain proteins. Based on the analysis available for fully sequenced genomes, mollusk genomes typically possess multiple genes encoding ShKT-domain containing proteins, for a global number that appears to be quite uniform across the three major molluscan classes, ranging from 15 in the limpet *Lottia gigantea* to nearly 50 in the oyster *Magallana gigas*. However, only a very few of these proteins has been functionally characterized so far as most of the sequences deposited in public databases derive from automated gene prediction. The two exceptions are Pf-ALMP, an astacin-like metalloproteinase highly expressed in the haemocytes of pearl oysters and implicated in proliferation processes [38], and a myc-like protein from *Crassostrea virginica*, tentatively linked to the control of cell cycle progression [39].

On the other hand, short secretory proteins containing a single ShKT domain have a very narrow taxonomical distribution. This simple architecture is typically found in the venom of sea anemones (Anthozoa). The only other animal phylum where a large number of ShKL sequences have been identified so far is Nematoda. Although most ShKL peptides from parasitic roundworms are simply known from automated gene predictions, some have attracted considerable interest due to their potential immunomodulatory properties [40]. Modica et al. have previously reported the presence of an ShKL peptide, produced with high salivary glands (SG) specificity, in *C. reticulata* [24]. Here, we expand the repertoire of ShKL proteins in this species, bringing their number to 19, most of which are characterized by a single ShKT domain, while five of them display multiple ShKT domains.

From an evolutionary point of view, ShKL genes are likely to derive from a lineage-specific innovation. ShKL genes are not present in the known genomes of gastropods and other molluscs, which possess a few genes encoding secretory proteins with multiple consecutive ShK domains associated with other domains, which will be discussed in detail below. The single exception, a protein from the freshwater snail *Biomphalaria glabrata* (XP_013076944.1) appears to be unrelated with those from *C. reticulata*. Hence, a common evolutionary origin for anthozoan, nematode and *Cumia* ShKL proteins is unlikely. Our phylogenetic reconstruction suggests that the *Cumia* ShKL sequences are part of a multigenic family, which evolved through a lineage-specific expansion event. Based on the placement of multiShKL 5 in the phylogenetic tree, and on the high conservation of its signal peptide compared to single-domain ShKL proteins, the currently expanded gene family might have been originated from an ancestral sequence with multiple ShKT domains. Only a more complete phylogenetic analysis including tissue-specific sequences from other gastropods could elucidate if the ancestral ShKL was SG-specific (the most parsimonious hypothesis according to our phylogeny), or instead lacked specificity, as it may be explained by a differential likelihood of the specificity loss/gain due to adaptive reasons (Figure 1, panels D and E).

Although the putative functional Lys-Tyr dyad in *Cumia* ShK, which corresponds to the one identified in most cnidarian ShK, is generally lacking (with the exception of CreShKL11), this does not imply a priori that these peptides lack any ion channel blocking activity. Indeed, the human matrix metalloprotease 23 in which Lys is replaced by Arg and Tyr by Leu still retains the ability to modulate Kv1 channels, albeit with a different specificity with respect to ShK [22]. Additionally, an ShKL peptide recently isolated from the sea anemone *Oulactis* sp., despite having a conserved dyad, is not active against K channels [41]. These observations indicate that while the presence of a dyad alone may not be sufficient for channel inhibition, alternative Lys or Arg residues of the ShK fold might have acquired the ability to block potassium channels, and different ShKT peptides may have evolved novel functional activities beyond potassium channel blockade. 

Whereas proteins having a CAP domain associated with ShKT are encoded by molluscan genomes, including the gastropods P. canaliculata [42], L. gigantea [43] and B. glabrata [44], none of these have been functionally characterized so far. However, two genes belonging to this family, Mr30 and Tex31, are highly expressed in the venom duct of cone snails, and even though the encoded proteins were originally connected to the proteolytic cleavage of conopeptide precursors, this claim was later dismissed [45,46]. The CAP domain is also associated with secretory proteins highly expressed in some ecdysozoan species. These include hymenopteran venom allergens [47] components of the salivary secretions of fleas [48] and other uncharacterized proteins from mosquitoes, ticks and mites. However, unlike vertebrate CRISPs and molluscan CAP-ShK proteins, none of these contains cysteine-rich C-terminal extensions.

Our phylogenetic analysis suggests an ancient evolutionary split between the two major clades of molluscan CAP-ShK and a possible evolutionary link between CreCAP-ShK type II proteins vertebrate CRISPs, and arachnids CAP-ShKT, while an ancient evolutionary connection between ecdysozoan venom allergens, nematodes CAP-ShK proteins and molluscan CAP-ShK type I genes cannot be excluded; however, it needs to be stressed that neither hymenopteran venom allergens nor the salivary proteins from arachnids contain ShK domains or other cysteine arrays (Figure 3). Hence, the most likely evolutionary scenario for this gene family includes the independent recruitment of ShKT modules in pre-existing ancestral CAP-domain containing proteins. Interestingly, the CAP domain, whose role has remained elusive for a long time, is now regarded as a cell surface anchor that is important for localizing and enhancing the action of CRISP domains, thanks to its lipid binding ability [49,50], which is possibly regulated through dimerization [51,52,53]. Variability in this domain may confer different membrane-binding properties, allowing a further diversification of molecular targets. For example, in tablysin (a salivary CAP protein produced by the horse fly *Tabanus yao*), this ability gave rise to a leukotriene scavenging activity that enables the inhibition of platelet aggregation [54]. However, in the light of our observations, CreCAP-ShK genes cannot be considered innovations of the *Cumia* lineage, as all molluscan CAP-ShK type I and II proteins are all inferred to derive from two common ancestral sequences, i.e., a prototypical CAP-ShK type I and a prototypical CAP-ShK type II gene. Conversely, the diversification and duplication of cysteine rich domains found in CreCAP-ShK type II domains seem to be a peculiar feature of *Cumia* lineage. Among them, the EGF domain has already been associated with toxin function: the omega-stichotoxin Sgt1a is a toxin with an EGF fold first isolated from the sea anemone *Stychodactyla gigantea* and initially named Gigantoxin-I [55]. Subsequently, similar toxins have been found in the sea anemones *Stychodactyla haddoni* [56], *Anemonia sulcata* and *Heteractis crispa* [57] among Cnidaria, and in the bloodworm *Glycera* [58]. Although it is not lethal, Sgt1a is able to induce tonic paralysis in crabs at low dose, and it also possesses a reduced but still detectable EGF activity on human cells, however, its potency is about 1000 times lower than human EGF [55]. In mammals, Sgt1a is able to elicit acute pain by indirect activation of the TRPV1 channel: the toxin binds to EGF receptors with its EGF domain, resulting in the activation of phospholipase A_2_ and the production of metabolites that activate the TRPV1 channel [59]. Although the pain induced by bloodworm bites is certainly an effective adaptation to deter predators [58], we presently have no evidence of a similar defensive function for the EGF domain of CreCAP-ShK type II peptides. 

Even though the function of CAP-ShK proteins in other molluscs is presently unknown, to evaluate the possibility of a feeding-related specialization we investigated their transcriptional patterns in the Pacific oyster *Magallana (= Crassostrea) gigas* [60] and in the golden apple snail *Pomacea canaliculata* [42]. In oysters, the single CAP-ShK type I gene was expressed at barely detectable levels in all tissues, whereas the CAP-ShK type II genes displayed mantle-specificity, indicating that *Cumia* CAP-ShK proteins, in spite of their origin from ancestral molluscan genes, underwent a switch in tissue specificity to the SG that might imply the acquisition of new feeding-related functions. In this framework, the repeated events of duplication and diversification of Cys-rich domains, including ShK and EGF, which seems to be a characteristic of the *Cumia* lineage may have evolved in order to allow a broader range of molecular targets. Variability of the ShK domain sequence, which has been convincingly shown to be responsible for ion channel regulation, has been related to differences in target specificity in snakes CRISPs [26]. 

In the absence of any functional characterization of CreCAP-ShK, we can only speculate that the high level of observed diversification may confer broad target specificity and multiple physiological roles for these proteins (especially for type II). These range from prey paralysis and anaesthesia to predator deterrence, and even immune response suppression, which could be achieved both via Kv1.3 channel modulation by the ShK domains and through leukotrienes scavenging by the CAP domain.

The M12 domain is associated with ShKT in several invertebrate taxa, including cnidarians, echinoderms and tunicates, even though such proteins have only been functionally characterized in the former phylum and associated with tissue morphogenesis, digestion and developmental processes [61,62]. As in the case of CAP-ShK proteins, parasitic worms (both Nematoda and Platyhelminthes) have developed an expanded repertoire of astacin-like proteins with ShKT domains [31]. In hookworms, they have been hypothesized to assist migration of infective larvae through host tissues and to play a fundamental role in the penetration of host mucosa by adult females [63]. M12-ShK genes are also present in several molluscan genomes, where they encode proteins with a variable number of cysteine-rich motifs. However, the only functionally characterized example remains Pf-ALMP, a protein highly expressed in the haemocytes of the pearl oyster *Pinctada fucata* and possibly involved in the regulation of cell proliferation [38]. While cnidarian and nematodes astacins generally possess one to three ShK domains, the presence of a higher number of ShK repeats is a trait that *Cumia* shares with other molluscs, (with up to 4 repeats found in *Octopus bimaculoides*, 8 in *Arion vulgaris* and 9 in *Pomacea canaliculata*) and with Platyhelminthes, with 5 repeats reported in the parasitic tapeworm *Schistocephalus solidus*. Our Bayesian analysis supports a high level of divergence of molluscan sequences from those found in cnidarians and nematodes, whereas those from flatworms are intermixed with those from gastropods and bivalves (Figure 3). This is congruent with the debated phylogenetic placement of Platyhelminthes, which are thought to be related to Lophotrochozoa according to the Platytrochozoa hypothesis [64]. Additionally, it suggests that the proliferation of ShK domains in M12-ShK proteins is a trait that may have convergently evolved in parasitic/predatory species of Lophotrochozoa. Predation is the primitive feeding habit of Cephalopoda like *O. bimaculoides*, and it is not infrequent in gastropods. In fact, although they belong to groups that are mostly omnivorous feeding preferentially on plant tissues, a predatory behaviour has been observed both in the apple-snail *P. canaliculata* and in *Arion* spp. For apple-snails predation on other species of freshwater snails has been reported [65], while different species of *Arion* slugs have even been observed feeding on young bird nestlings [66]. 

Remarkably, CreM12-ShK 4, the only protein containing just 3 ShKT domains and also one of the two to lack strong SG-specificity, was clearly related to orthologous metalloproteases found in all molluscs, regardless of the feeding habits of the species of origin. The inclusion of pearl oyster metalloproteases (Pf-ALMP) in the same clade indicates that these proteins may share an ancestral function in the regulation of cell proliferation. On the other hand, the grouping of the other six M12-ShK in a strongly supported monophyletic clade together with the lack of tissue specificity of the orthologous genes from the other molluscs included in our phylogenetic reconstruction, points towards a common origin of these proteins that subsequently were duplicated in the salivary glands of the *Cumia* (or Buccinoidea) lineage. Their functional significance may be supported by the apparent lack of tissue specificity of the orthologous genes from the oyster *M. gigas* and the apple-snail *P. canaliculata*, which were expressed at variable levels in all tissues. It should also be noted that a fibrino(geno)lytic activity has been reported for spider venom astacins [67], suggesting that these proteins might contribute to hemostasis impairment in hematophagous snails.

## 4. Conclusions

Summarizing our results, we observed a remarkable level of both domain and gene duplication in *C. reticulata* ShKT-containing proteins. Domain repeats are widespread in eukaryotic genomes where they are thought to arise via tandem duplications within a gene, which may involve single domains or units containing several repeated domains, thus increasing the rate of generation of multiple repeats [68,69,70,71]. In addition to these internal duplications, the presence of multiple paralogous copies for all *C. reticulata* ShK genes is in line with the widespread presence of lineage-specific expanded gene families in eukaryote genomes [72], which are often linked with highly specialized functions in molluscs [73,74]. The number of domain repeats is often extremely variable in orthologous genes, indicating that the loss/gain of domains in repeats is frequent in evolution [75]. Additionally, variations in the number of extracellular domains such EGF have been explained by exon shuffling mechanisms [76]. In *C. reticulata* ShKT containing proteins, the recurrent domain and gene duplication that was observed are likely to guarantee a high level of functional diversification. Given the detected signature of both tissue and lineage specific gene duplication (with the exception of CreCAP-ShKT), we can confidently hypothesize that these proteins may play a key role in feeding, as previously reported for the cytolytic coluporins [35]. The sequence variability of the multiple ShK domains may confer a broad target specificity and the ability to modulate several different types of ion channels. 

Overall, our results seem to indicate that the potentially increased functional versatility offered by domain and gene duplications may be instrumental to the ecological needs of *C. reticulata*, thus, broadening the taxonomic spectrum of the potential hosts, and providing the remarkable advantages that such a generalist feeding habit may offer to a slow-moving parasite feeding on fast-moving hosts. 

## 5. Materials and Methods

### 5.1. Identification and Characterization of ShKT Domain Containing Protein Sequences

The translated ORFs derived from *C. reticulata* salivary, mid-oesophageal and whole-body transcriptomes [24], assembled de novo using different methodologies as described in Gerdol et al. 2018 [35], were analyzed with Hmmer v.3.1b2 [77] to detect significant matches with the ShKT domain. Compared to the previous work [35], protein predictions were obtained using a lower minimum ORF length threshold, equal to 50 codons, taking into account the average expected length of single-domain ShK-like proteins. ShkT-domain containing proteins were identified by the detection of the conserved domain based on the Pfam PF01549 signature with e-value < 0.05.

The completeness and correctness of the obtained protein sequences and their encoding mRNAs were evaluated by the assessment of a uniform sequencing coverage, as previously described [35]. Validated sequences were used as queries in tBLASTn searches against the transcriptomes with the aim of detecting possible additional matches, characterized by the presence of non-canonical ShKT-like domains, whose score initially fell below the Hmmer threshold. The reliability of newly obtained sequences was evaluated as described above and the process was recursively repeated until no more hits could be identified.

All *C. reticulata* complete and tissue specific (see the next section) ShKT domain containing proteins were subject to analysis with Phobius [78] and WoLF PSORT [79] to detect the presence of signal peptide regions for secretion, alpha-helical transmembrane domains and to infer their subcellular localization. InterProScan v.5 [80] was used to annotate additional conserved domains associated with ShKT in multidomain proteins.

All ShKL, multiShKL, CreCAP-ShK and M12CAP-ShK sequences were deposited in the NCBI GenBank repository under the accession IDs MK387102-MK387134.

### 5.2. Calculation of Gene Expression Values

To ensure broad comparability both within and between samples, the expression level of *C. reticulata* ShKT-domain containing transcripts are here reported as transcripts per million (TPM), calculated using the formula described by Wagner and colleagues [81]. Namely, sequencing data from Modica et al., 2015, linked to three tissues (salivary gland, mid-oesophageal gland and the whole body except for the two aforementioned organs), with three biological replicates each, were used to infer gene expression values. Trimmed reads were mapped on the non-redundant *C. reticulata* transcriptome with high stringency (length fraction = 0.75, similarity fraction = 0.98) using the *map reads to reference* tool included in the CLC Genomics Workbench v.11 (Qiagen, Hilden, Germany). We used Log-transformed TPM values to generate heat maps for graphically depicting the transcriptional profiles of ShKT-domain containing proteins across tissues.

The expression levels of target orthologous genes were computed with the same strategy in *Pomacea canaliculata* [42] (in gills, lung, ovary, testis, haemocytes, stomach, albumen gland, kidney, mantle and digestive gland) and *Magallana gigas* [60] (inner and outer mantle, male and female gonad, digestive gland, gills, labial palps, posterior adductor muscle and hamocytes). The RNA-seq datasets analyzed were retrieved from the NCBI SRA database (BioProjects PRJNA427478 and PRJNA423172 for *P. canaliculata*, PRJNA146329).

### 5.3. Phylogeny of C. reticulata ShKT-Domain Containing Proteins

The protein sequences identified as described above, were divided into three different subgroups: (i) single domain ShK-like proteins; (ii) proteins with an ShKT domain, associated with cysteine-rich secretory proteins, antigen 5, and pathogenesis-related 1 proteins (CAP) domains, identified by the presence of the IPR014044 signature; and (iii) proteins with an ShKT domain associated with a Peptidase M12A domain, identified by the presence of a IPR001506 signature.

The three sequence sets were independently subjected to multiple sequence alignment with MUSCLE [82], together with other metazoan sequences chosen in order to maximize both functional and taxonomic coverage (see below for details). In all analyses, sequences derived from the genomes of *Pomacea canaliculata* [42], *Lottia gigantea* [43] and *Biomphalaria glabrata* [44], were selected as representatives for Caenogastropoda, Patellogastropoda and Heterobranchia, respectively. Multiple alignments used for phylogenetic reconstruction are available as Supplementary Material (Text S1).

The phylogeny of CreCAP-ShK proteins was built using the region corresponding to the CAP domain only, due to the heterogeneous organization of the C-terminal cysteine-rich domain. Representative vertebrate CRISPs, ecdysozoan venom allergens and salivary gland secretory proteins, the *Conus* proteins Mr30 and Tex31 and a putative venom protein from the blue ringed octopus *Hapalochlaena maculosa* [37] were also included in the multiple sequence alignment. We evaluated the WAG model of molecular evolution, with a proportion of invariable sites and a gamma-distributed rate variation across sites, as the best-fitting one for this dataset [83], using ModelTest-NG (https://github.com/ddarriba/modeltest/). For M12-ShK proteins, phylogenetic inference was carried out on the region corresponding to the astacin domain only. Representative sequences from cnidarians (based on the *N. vectensis* genome), nematodes (including those derived from the genome of *C. elegans*) and Platyhelminthes were included in the MSA, together with Pf-ALMP from the pearl oyster *P. fucata* [38]. All the sequences included displayed the characterizing M12 domain accompanied by one or more ShKT domains. In this case, the selected model for molecular evolution was VT+I+G.

Phylogenetic inference was performed in both cases with MrBayes 3.2.1 [84] running two independent MCMC analysis in parallel with four chains each, for 200,000 generations. Run convergence was evaluated with Tracer v.1.7.1 (https://github.com/beast-dev/tracer/) based on achieving an effective sample size value >= 200 for all the estimated parameters. The first 25% of sampled trees was discarded with the burnin procedure, and the remaining trees were used to compute a consensus tree.

## Supplementary Materials

The following are available online at http://www.mdpi.com/2072-6651/11/2/106/s1, Text S1: Multiple alignments used for phylogenetic reconstruction.
